# Psychometric properties of the environmental factors’ questionnaire for undergraduate medical students taking online learning during COVID-19 pandemic: a cross-sectional study

**DOI:** 10.1186/s12909-023-04314-0

**Published:** 2023-05-22

**Authors:** Ola K. Taleb, Ab Hamid Siti-Azrin, Abdullah Sarimah, Ali H. Abusafia, Kamarul Aryffin Baharuddin, Wan Adnan Wan-Nor-Asyikeen

**Affiliations:** 1 Biostatistics and Research Methodology Unit, School of Medical Sciences, Universiti Sains Malaysia, Kubang Kerian, Kelantan, 16150 Malaysia; 2 School of Health Sciences, Universiti Sains Malaysia, Kubang Kerian, Kelantan, 16150 Malaysia; 3 Department of Emergency Medicine, School of Medical Sciences, Universiti Sains Malaysia, Kubang Kerian, Kelantan, 16150 Malaysia; 4 Hospital Universiti Sains Malaysia, Kubang Kerian, Kelantan, 16150 Malaysia

**Keywords:** Environmental factors, Online learning, Medical students, Validity, Reliability, COVID-19

## Abstract

**Background:**

Environmental factors are important for students’ learning during online classes, especially during a pandemic, such as COVID-19. This study aimed to validate the environmental factors’ questionnaire during online learning.

**Methods:**

A total of 218 undergraduate medical students at the Health Campus, Universiti Sains Malaysia, participated in a cross-sectional study that involved an online survey. Environmental factor scales were assessed with the nine-item lighting, noise, and temperature (LNT) scale and the six-item technology scale. Analysis was performed using confirmatory factor analysis (CFA).

**Results:**

The English version of the LNT scale with nine items and three factors showed a good fit to the data, with no item deleted. For LNT, the composite reliability (CR) was 0.81, 0.81, and 0.84, respectively, while the average variance extracted (AVE) was 0.61, 0.59, and 0.6, respectively. The English version of the technology scale, with six items and one factor, also showed a good fit to the data, with no item deleted. The CR was 0.84, and the AVE was 0.51.

**Conclusions:**

The results provide psychometric evidence for environmental questionnaire scales in evaluating the factors associated with online learning among Malaysian university medical students. All items were retained and confirmed to fit the sample data.

## Background

The novel coronavirus-2 (SARS-Cov-2) resulted in the closure of schools and institutions, as well as the suspension of face-to-face learning and teaching activities. Since movement restriction orders and campus closures had an impact on formal learning, online learning was selected as the best choice for continuing the learning process [1].

Online learning, which is conducted in synchronous and asynchronous settings using a variety of devices with internet connections, including PCs, laptops, tablets, and mobile phones, has become an alternative learning method [2]. Students are required to adjust to new environmental factors, such as temperature, noise, lighting, and technology, that are different from those in the classrooms at their university [3]. These new environmental factors might be distressing and affect the way students study in an online class.

Natural lighting is synonymous with daylight, which occurs when light is transferred through sunshine, reflected on a surface, and subsequently illuminates an area or place. In most buildings, daylight is a helpful light source, particularly in learning environments such as classrooms, where the quality of natural light is superior to that of any artificial lighting. Artificial lighting is required to create a safe and suitable learning environment for students [4]. There is evidence that the quality of room lighting impacts students’ learning [5].

A significant environmental issue is noise pollution. It is described as an undesired sound that could have negative impacts on a person’s physical health (like hearing loss) and psychological health (like annoyance and frustration) [6]. Excessive noise is detrimental to the teaching–learning process because it distracts and limits attention and cognition [7, 8].

A comfortable temperature is described as a mental state that conveys satisfaction with the thermal environment [9]. An uncomfortable temperature causes dissatisfaction and unhappiness among students, thus affecting their productivity [10].

The technological factor is one of the instrumental aspects influencing the success of online education [11]. Online technology can improve learning by being more productive than what is done in person or through other methods [12]. The online learning process is more difficult due to inadequate online learning infrastructures and limited internet accessibility for students [13].

From the literature, the Questionnaire of Effects from Online Classes (QEOC) on University Students’ Health and Academic Performance is a possible tool to measure environmental factors, such as lighting, noise, and temperature (LNT) [3]. It is a straightforward, useful, and efficient tool for evaluating the attributes of environmental factors, and it has good validity and reliability in measuring and analyzing environmental factors [3]. Validity is defined as the ability of a tool to measure the attributes that are supposed to be assessed, and reliability is the consistency or reproducibility of measurements over time or on different occasions [14, 15]. The Cronbach’s alpha coefficient values of the three domains (LNT) were more than 0.7 [3].

A student satisfaction survey form (SSSF) is an instrument to measure students’ satisfaction in online learning environments. It consists of five domains: instructor, technology, class management, interaction, and instruction [16]. The reliability of Cronbach’s alpha coefficient for each domain was 0.75, 0.84, 0.70, 0.57, and 0.80, respectively [16].

**Fig. 1:**
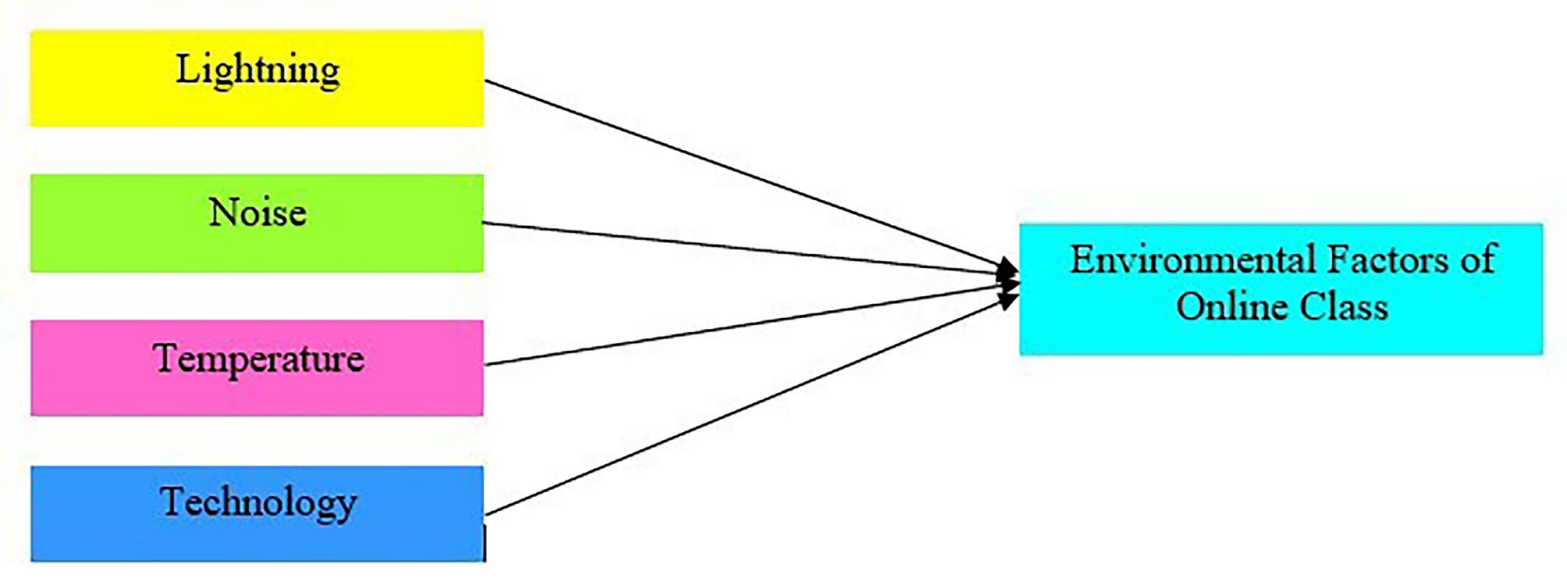
Conceptual framework of lighting, noise, temperature (LNT), and technology that affects the online learning of students

The purpose of this study was to assess the validity and reliability of environmental questionnaires, which consist of lighting, noise, temperature (LNT), and technology that affects the online learning of students. The conceptual framework is illustrated in Fig. 1.

## Materials and methods

### Study design and ethical clearance

A cross-sectional study involved online survey was conducted among 218 undergraduate medical students in Universiti Sains Malaysia (USM). They voluntarily completed the survey. Ethical clearance was conducted from the Human Ethics Committee USM.

### Sample size and sampling method

The sample size for confirmatory factor analysis (CFA) was estimated based on Kline [17]. Two hundred and fifty students were the minimum sample size needed for the study, with a 20% dropout rate considered. A convenience sampling method was applied to collect the participants who fulfilled the inclusion and exclusion criteria.

The inclusion criteria were fourth- or fifth-year medical students during the 2021/2022 academic sessions, who were able to read and understand the English language, and who were involved with online learning during the second semester of the 2020/2021 academic session. The exclusion criteria were re-sit students.

### The environmental questionnaire

The environmental questionnaire consists of two scales: the LNT scale and the technology scale. Permission to use the questionnaire was obtained from the authors. The LNT scale is a nine-item scale that was adapted from the QEOC. It was developed and validated by Realyvásquez-Vargas et al. (2020) [3]. It measures the environmental factors that impact students’ online classes in three domains: lighting, noise, and temperature. The tool uses a five-point Likert scale, where 1 = Never, 2 = Hardly ever, 3 = Sometimes, 4 = Usually, and 5 = Always. The first domain is lighting (three items), which measures the effects of indoor lighting on students’ online classes. The second domain is noise (three items), which measures the effects of noise pollution’s effects on students’ online classes. The third domain is temperature (three items), which measures thermal comfort effects on students’ online classes.

The technology scale is a six-item scale that was adapted from the SSSF. It was developed and validated by Abou Naaj et al. (2012) [16]. It measures the adequacy of technology that impacts students’ online classes. It uses a five-point Likert scale, where 1 = Strongly disagree, 2 = Disagree, 3 = Neither agree nor disagree, 4 = Agree, 5 = Strongly agree.

### Data analysis

CFA investigated the internal structure of the LNT and technology scales using R Studio software version 3.6.0. The latent constructs of the LNT and technology scales were evaluated using absolute fit indices (i.e., root mean square error of approximation (RMSEA) and standardized root mean square residual (SRMR)) and incremental fit indices (i.e., comparative fit index (CFI) and Tucker-Lewis index (TLI)).

Standardized factor loading (SFL) is the contribution of observed variables to respective latent variables. High loading indicates a high contribution of the item to the domain. An SFL of more than 0.5 is considered an acceptable value [18]. Modification indices (MIs) are diagnostic statistics for initial models that enhance poor model fitness to capture model misspecification [19]. When there are numerous high MI parameters, the starting point should be the greatest MI. Any model modification must be justified by both theoretical and empirical evidence [20]. Model-to-model comparison, Aikaike information criterion (AIC), and Bayesian information criterion (BIC) are commonly used when comparing non-nested models estimated with the same data to reveal the models that are the most parsimonious. Smaller AIC and BIC values signify a better model fit.

Composite reliability (CR) was used to estimate the reliability of the LNT and technology scales, with 0.6 and above the minimum acceptable range for CR [21]. The average variance extracted (AVE) results were evaluated for convergence validity. AVE must fall within the range of 0.5 or higher [22]. When the items from different factors have a weak correlation with one another, discriminant validity exists [18]. Since there was a negative correlation (r) between the factors, discriminant validity was confirmed [17].

## Results

### Demographic characteristics

Table 1 provides information about the participants’ demographics. Out of the 218 participants, 72.9% were female, and 27.1% were male. Most participants were Malay (78.4%) and stayed on campus (95.4%). About 57.8% of the participants were from Year 4. Family income for most of the participants was below the bottom 40% household income range, which was less than RM4850 (42.7%). Based on the Department of Statistics Malaysia 2020, a household with a monthly income under the middle 40% is between RM4,851 and RM10,970, and the top 20% is more than RM10,971. Most of the participants used wi-fi for online classes (90.8%). The laptop was the most common digital tool used by the participants (46.8%).

**Table 1:** Demographic characteristics of the participants (n = 218)

Characteristics		n (%)
Gender	Female	159 (72.9)
	Male	59 (27.1)
Ethnicity	Malay	171 (78.4)
	Chinese	15 (6.9)
	Indian	19 (8.7)
	Others	13 (6.0)
Academic years	4th year	126 (57.8)
	5th year	92 (42.2)
Family income (RM)	≤ 4850	93 (42.7)
	4851–10,970	49 (22.5)
	> 10,971	76 (34.9)
Mode to access online classes (most of use)	Wi-fi	198 (90.8)
	Mobile data	19 (8.7)
	Internet cafe	0 (0.0)
	Wi-fi and mobile data	1 (0.5)
Current accommodation	Inside campus	208 (95.4)
	Urban	6 (2.8)
	Rural	4 (1.8)
Digital tools	Laptop	102 (46.8)
	Mobile phone	31 (14.2)
	I-pad/Tablet	76 (34.9)
	Desktop	9 (4.1)

### LNT scale

Table 2 lists the summary of the nine items of the LNT scale.

**Table 2:** Summary of nine-item characteristics for LNT (n = 218)

Items	Score, n (%)				
	Never (1)	Hardly ever (2)	Sometimes (3)	Usually (4)	Always (5)
Q1: The level of lighting in my study area allows me to see clearly what is around.	0 (0.0)	1 (0.5)	33 (15.1)	77 (35.3)	107 (49.1)
Q2: I can control the level of lighting in my study area when taking online classes	0 (0.0)	14 (6.4)	23 (10.6)	68 (31.2)	113 (51.8)
Q3: The level of lighting (from lamps, computer screen) in my study area allows me to have visual comfort	2 (0.9)	3 (1.4)	25 (11.5)	86 (39.4)	102 (46.8)
Q4: I have privacy in my study area when taking classes online	19 (8.7)	34 (15.6)	64 (29.4)	47 (21.6)	54 (24.8)
Q5: The noise level (coming from devices, people’s talks, external sources) in my study area allows me to concentrate	10 (4.6)	31 (14.2)	73 (33.5)	62 (28.4)	42 (19.3)
Q6: I can control the noise level in my study area	17 (7.8)	31 (14.2)	58 (26.6)	63 (28.9)	49 (22.5)
Q7: The temperature in my study area allows me to be comfortable and concentrate	3 (1.4)	11 (5.0)	51 (23.4)	91 (41.7)	62 (28.4)
Q8: I can control the temperature in my study area	7 (3.2)	19 (8.7)	45 (20.6)	78 (34.8)	69 (31.7)
Q9: The air quality in my study area is appropriate	2 (0.9)	7 (3.2)	35 (16.1)	91 (41.7)	83 (38.1)

### LNT measurement model

**Fig. 2:**
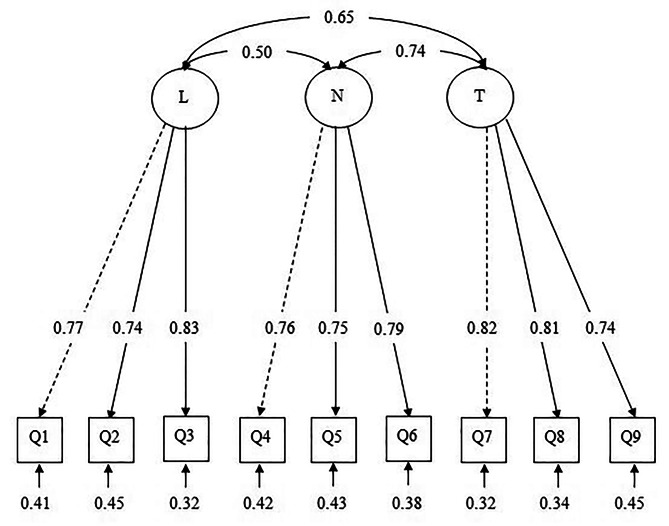
Factor loading of LNT domains based on the final model

The initial hypothesized model (Model 1) estimated using the maximum likelihood estimator (MLR) comprised nine items with three factors. The results of Model 1 showed good fit indices with CFI = 0.99, TLI = 0.98, SRMR = 0.03, and RMSEA (90% CI) = 0.03 (0.00, 0.07). All factor loadings were between 0.74 and 0.82 (Fig. 2). The final model was then evaluated for CR and AVE. Lighting had a CR of 0.82, noise of 0.81, and temperature of 0.84. The AVE for LNT were 0.61, 0.59, and 0.63, respectively. The r between lighting and noise was 0.51, p-value < 0.001, lighting and temperature was 0.65, p-value < 0.001, and noise and temperature were 0.74, p-value < 0.001. Although r was significant, it was less than 0.85, demonstrating that the three factors have good discriminant validity. Table 3 displays the results for CR and AVE.

**Table 3:** Factor loadings, composite reliability and average variance extracted of the LNT measurement model

Factor	Item	Factor loading	CR	AVE
Lighting	Q1	0.77		
	Q2	0.74	0.82	0.61
	Q3	0.82		
Noise	Q4	0.76		
	Q5	0.75	0.81	0.59
	Q6	0.79		
Temperature	Q7	0.82		
	Q8	0.81	0.84	0.63
	Q9	0.74		

### Technology scale

Table 4 lists the six items that were applied to assess the technical quality of online classes.

**Table 4:** Summary of item characteristics for technology (n = 218)

Items	Score, n (%)				
	Strongly disagree (1)	Disagree (2)	Neither agree nor disagree (3)	Agree (4)	Strongly Agree (5)
Q10: The instructor’s voice is audible	0 (0.0)	5 (2.3)	34 (15.6)	135 (61.9)	44 (20.2)
Q11: Course content shown or displayed on the smart board is clear	1 (0.5)	4 (1.8)	23 (10.6)	135 (61.9)	55 (25.2)
Q12: The microphone is in good working condition	0 (0.0)	6 (2.8)	29 (13.3)	133 (61.0)	50 (22.9)
Q13: The video image is clear and comprehensive	2 (0.9)	9 (4.1)	31 (14.2)	130 (59.6)	46 (21.1)
Q14 Technical problems are not frequent, and they do not adversely affect my understanding of the course	8 (3.7)	23 (10.6)	52 (23.9)	106 (48.6)	29 (13.3)
Q15: The technology used for online teaching is reliable	3 (1.4)	2 (0.9)	27 (12.4)	133 (61.0)	53 (24.3)

### Technology measurement model

**Fig. 3:**
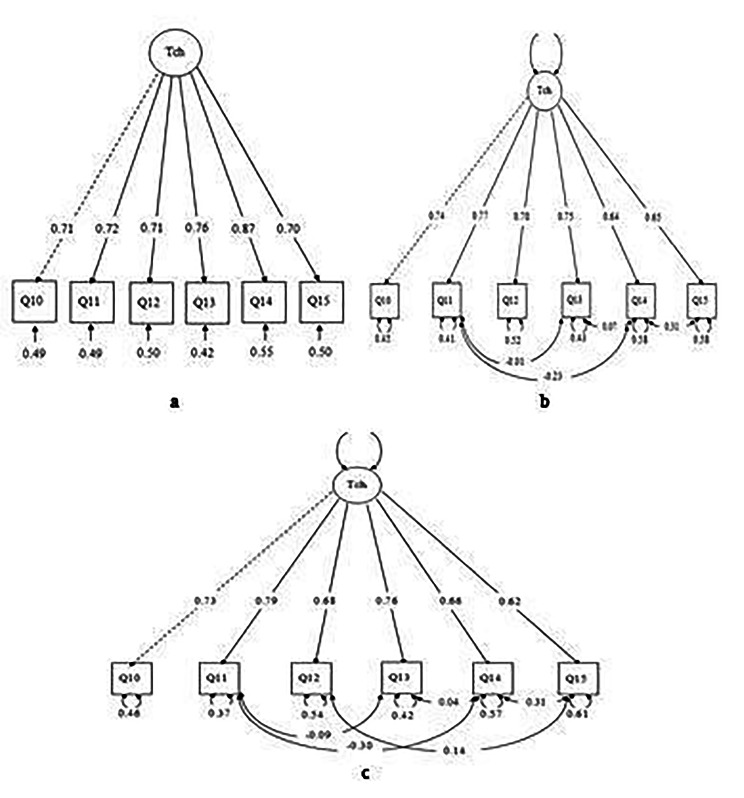
(**a**) Factor loading of technology based on Model 1; (**b**) Factor loading of technology based on Model 2; (**c**) Factor loading of technology based on Model 3

The initial hypothesized model estimated using MLR comprised six items with only one factor. The initial measurement model (Model 1) robust fit indices of RMSEA were more than the maximum recommended value of 0.08, while TLI and CFI were less than the minimum recommended value of 0.95, as summarized in Table 5. All factor loadings ranged from 0.67 to 0.76 (Fig. 3).

Then, the items with correlated residuals for Q14 with Q11, Q13 with Q15, and Q13 with Q11 were added in a subsequent investigation to improve the initial model. The findings of the second model (Model 2) revealed a good model fit based on all indices, except for the upper 90% CI of robust RMSEA = 0.15, with CFI = 0.98, TLI = 0.96, SRMR = 0.03, and RMSEA = 0.07 (Table 5). All factor loadings were between 0.64 and 0.77 (Fig. 3).

Further adjustment was performed by adding items with correlated residuals for Q15 with Q12 to enhance the second model. The results of the third model (Model 3) revealed a good model fit based on all indices, except for the upper 90% CI of robust RMSEA = 0.16. CFI = 0.99, TLI = 0.97, SRMR = 0.02, and RMSEA = 0.06 (Table 5). All factor loadings were between 0.62 and 0.79 (Fig. 3).

The standardized item loading for the three Technology-M models is shown in Fig. 3. All the parameter estimates were acquired from the original main hypothesized measurement models in Table 6. The standard item loading ranged from 0.67 to 0.76, 0.64 to 0.77, and 0.62 to 0.79, respectively, according to the results of Models 1, 2, and 3, which are considered to have good to excellent factor loading. The three models were compared using AIC, BIC, and X^2^ difference. Model 3 was chosen as the best and final model based on the smallest AIC, BIC, and significant difference between the model and its value for the better fit indices (Table 5).

The final model was then evaluated for CR and AVE and was noted at 0.84 and 0.51, respectively. The construct validity of the factor was considered good. The results for the CR and AVE technology models are presented in Table 6.

**Table 5:** Model fit indices for technology measurement model

Model	CFI	TLI	SRMR	RMSEA (90%CI)	AIC	BIC
Model-1	0.94	0.90	0.05	0.122 (0.066, 0.181)	2438.4	2495.9
Model-2^a^	0.98	0.96	0.03	0.073 (0.000, 0.157)	2440.0	2494.1
Model-3^b^	0.99	0.97	0.02	0.065 (0.000, 0.164)	2438.4	2495.9

^a^Model with correlated item residual of Q14 with Q11, Q13 with Q15, and Q13 with Q11

^b^Model with correlated item residual of Q14 with Q11, Q13 with Q15, Q13 with Q11, and Q15 with Q12

**Table 6:** Factor loadings, composite reliability and average variance extracted of technology measurement model

Factor	Item	Factor loading	CR	AVE
Technology	Q10	0.73	0.84	0.51
	Q11	0.79		
	Q12	0.67		
	Q13	0.76		
	Q14	0.65		
	Q15	0.62		

## Discussion

This study assessed the validity and reliability of the environmental questionnaire (LNT and technology) scales among 218 undergraduate medical students at USM using CFA. The results showed acceptable evidence of the validity and reliability of the LNT and technology scales. The LNT and technology scales fit the data well and provided strong evidence of the construct validity of the scales. The MLR estimator was employed, as the multivariate normality assumption was violated.

The LNT model was confirmed by the CFA model. It was hypothesized to contain nine items in a three-factor model. The initial model provided a good fit for the data. Thus, there was no need for modification. Construct validity using convergent validity (AVE and CR) and discriminant validity were applied to the LNT model. LNT had estimated AVEs of 0.61, 0.59, and 0.63, respectively. All of these values were greater than the recommended value of 0.5 [22]. Meanwhile, the construct reliability using CR was 0.82, 0.81, and 0.84 for LNT, respectively, and it was higher than the recommended level of 0.60 [21]. Moreover, the correlation between lighting and noise was 0.51, lighting and temperature was 0.65, and noise and temperature were 0.74 in the LNT model, which was less than the recommended value of 0.85 [18]. Therefore, these three LNT subscales are distinct, and each factor contributes to the explanation of a different variance from the others. The final measurement model for the LNT English version (LNT-M) tested in this study is consistent with Realyvásquez et al. (2020) [3], as all the items were kept and confirmed to be fit for the sample data.

Regarding the technology model, CFA was conducted on one factor with six items. The initial model did not achieve a good fit for the data. Thus, modification was done to improve the model using MIs by correlating the residual for Q14 with Q11, Q13 with Q15, and Q13 with Q11. The results of the second model (Model 2) showed a good model fit based on all indices, despite the upper 90% CI of robust RMSEA being 0.15. Further modification with a correlated residual for Q15 with Q12 was added to the second model. The results of the third model (Model 3) showed a good model fit based on all indices, except for the upper 90% CI of robust RMSEA = 0.16. Hu and Bentler (1998) [23] reported that the high RMSEA value tends to over-reject when the sample size is small (n = 250). According to Kenny et al. (2015) [24], it was suggested not to calculate the RMSEA for small df models, particularly those with small sample sizes. Therefore, the acceptance of the model was based on CFI and SRMR. However, in the current study, the estimated RMSEA, CFI, TLI, and SRMR are good (CFI = 0.99, TLI = 0.97, SRMR = 0.02, RMSEA = 0.06). Therefore, Model 3 was accepted.

After developing the technology model, the researcher further examined its construct validity based on convergent validity (AVE and CR). The estimated AVE for the technology was 0.51 above the recommended value of 0.5 [22]. The construct reliability using CR was 0.84, exceeding the recommended level of 0.60 [21]. The final measurement model for the technology English version tested in this study is similar to Abou et al. (2012) [16] and Selvanathan et al. (2020) [25].

### Limitations

Despite having a valid and reliable model, there are still several shortcomings that can be considered for future research. For this study, only one medical school participated. The results may not be applicable to other medical schools or institutes. Multi-center research is recommended to verify the current findings. Also, rather than asking participants about their experiences this semester, this questionnaire asked them about the semester prior. Therefore, it might not completely reflect the respondents’ judgments about environmental factors. Purposive sampling is also better for CFA, since it allows a researcher to select cases that adhere to the study’s guidelines. However, convenience sampling was chosen over purposive sampling due to its lower costs, simplicity, and time savings. Finally, due to COVID-19 status, an online survey was used to collect data rather than in-person interviews.

## Conclusion

The environmental questionnaire exhibited a satisfactory level of construct validity and a high level of reliability, making it suitable for use in a medical school setting to evaluate environmental factors pertinent to LNT. Therefore, the LNT and technology questionnaire is a valid and reliable psychometric property for assessing the environmental factors during online learning for undergraduate medical students.

## Data Availability

The datasets generated and/or analysed during the current study are not publicly available but are available from the corresponding author on reasonable request.
